# Evaluation of the Postoperative Nursing Effect of Thoracic Surgery Assisted by Artificial Intelligence Robot

**DOI:** 10.1155/2021/3941600

**Published:** 2021-11-16

**Authors:** Xiufen Hu, Xiaodan He

**Affiliations:** No. 1 Department of Thoracic Surgery, Liaoning Cancer Hospital, Shenyang, Liaoning 110042, China

## Abstract

In order to evaluate the postoperative nursing effect of artificial intelligence robot-assisted thoracic surgery, this study proposed the Da Vinci robot-assisted pulmonary lobotomy, from January to December 2014; 42 patients (15 males and 27 females, aged 33–69 years old) underwent lobectomy with the Da Vinci robot system in the chest hospital. A series of postoperative nursing was carried out. The surgical results showed that 42 patients with Da Vinci robot-assisted lobectomy had operation time of 62–225 min and blood loss of 70–300 mL. There was no intraoperative blood transfusion, the intraoperative central rate was maintained at 60–100 times/min, and the blood pressure was maintained at 90–140/60–90 mmHg. No patient was transferred to thoracotomy, and 2 patients were performed robotic wedge resection first, and then, robotic lobectomy was performed after malignant tumor was confirmed by freezing results, with relatively light postoperative pain, no infection, beautiful wound, and smooth recovery and discharge. Robot-assisted lobectomy is a new technique with advantages of less trauma, less pain, faster recovery, and safer and more thorough lymph node dissection.

## 1. Introduction

Cancer is the main cause of death. Lung cancer, as the most common cancer, develops rapidly and has a great impact on the quality of life of patients. Therefore, timely and effective treatment of lung cancer is extremely important [1]. With the development and progress of modern science and technology, many therapeutic techniques are changing. Taking nonsmall cell lung cancer as an example, the most effective treatment is surgical resection of the focal tissue. From the initial thoracotomy to the minimally invasive revolution in 1980s, more and more studies have shown that minimally invasive surgery has great advantages in the survival rate and long-term efficacy of lung cancer patients [2]. The application of surgical robots in the 21st century has become a new opportunity in the history of minimally invasive surgery. The use of robots, however, remains controversial. Proponents emphasize its superior 3D imaging, maneuverability, and technical advantages over conventional thoracoscopy, such as motion-scaling and vibration-filtering capabilities [3]. The Da Vinci robotic surgical system is a new interdisciplinary technology combining robotics, computer technology, digital image processing technology, micromotor system, sensor technology, biological manufacturing, and clinical technology. It is the first robotic system used in thoracic surgery. This technology has the advantages of high accuracy, small trauma, light postoperative pain, quick recovery, and beautiful wound, and its clinical application brings a new mode of surgical nursing cooperation to the operating room nursing work [3]. Da Vinci Robotic Surgical Inc. (Intuitive Surgical Inc. and Mountain View Intuitive) CA was composed mainly of a surgeon console, a PatientCart with four 7-dOF interactive arms, and a high accuracy 3DHD vision system (VisionCart). The console composed of the computer system, surgical operation monitor, robot control monitor, operation handle, and input and output devices [4]. During surgery, the surgeon sits at a console away from the operating table, his head resting on a field frame, and his eyes receive the full image from different cameras to synthesize a three-dimensional image of the surgical field. The physician controls the operating lever with both the hands, and the hand movements are conveyed to the tip of the mechanical arm to complete the surgical operation [5]. Opponents say the technology is immature, and the machines unstable and expensive. At present, the robot has been applied in many surgeries, such as urology, obstetrics, and gynecology, and general surgery Robot surgery has been reported in thoracic surgery, but its safety, advantages, and disadvantages are controversial. In view of this research problem, Siu et al. found that the proportion of patients with blood loss, lung infection, and pneumothorax in the RATS group was lower than that in the VATS group, which was related to less injury during the robot operation. However, RATS have a higher incidence of arrhythmia, which may lead to reduced injury and physiological pressure during operation with robots, resulting in reduced catecholamine secretion and increased risk of arrhythmia. More complications of robots will not affect the recovery speed and quality of postoperative patients [6]. Zoumprouli et al. in their study observed that people think that the robot of artificial intelligence such as the surgical system provides high-resolution three-dimensional images, its unique eye tracker can accurately track the physician's line of sight, the sophisticated software is to produce the corresponding organization of 3D images, and the movement of the image into A static picture, this means that when the robot devices move up and down in the body, what surgeons see is still a still picture, reducing the interference of surgery [7]. Obuchi et al. have found that the artificial intelligent robotic surgical system can perform whole resection of superior mediastinum fat and lymphatic tissue, and the degree of thorough dissection is even higher than that of traditional thoracotomy due to clear visual field and delicate endowrist movements. With the help of the robot's delicate arm and instruments, the ligation of traditional surgical vascular sheath line can be accomplished very lightly under the microscope, which can greatly save the cost of traditional endoscopic surgical instruments [8]. On the basis of the current study, this study puts forward the Da Vinci robot-assisted lung resection, the method using, in December, 2014, thoracic hospital application of the Leonardo Da Vinci robot system line, 42 cases of lung resection, 15 cases were male, female 27 cases, aged 33–69 years old, and a series of postoperative nursing; the surgical results showed that in 42 cases of Da Vinci robot-assisted lobectomy patients, the operative time was 62–225 min, the blood loss was 70–300 ml, no intraoperative blood transfusion, the operative center rate was maintained at 60–100 times/min, the blood pressure was maintained at 90–140/60–90 mmHg, and no patient was converted to thoracotomy. Two of the patients underwent robotic wedge resection and waited for the freezing results to confirm the malignant tumor before robotic lobectomy. After surgery, all patients woke up in the anesthesia recovery room. After waking up, all indicators were qualified (normal blood gas indicators, restored muscle tension, and protective reflex), and vital signs were stable; they returned to the intensive care unit (ward). The mean postoperative hospital stay was (5 ± 2.5) d. The average indwelling time of chest tube was (4.0 ± 1.9) d. 24 h after surgery, the average volume of chest drainage was (200.4 ± 187.7) mL. Postoperative pain was relatively light, no wound infection, beautiful, all recovered, and patients were discharged successfully. Robot-assisted pulmonary lobectomy is a new technology with advantages of less trauma, less pain, faster recovery, and safer and more thorough surgical lymph node dissection [9].

## 2. Objects and Methods

### 2.1. Object

**Figure 1:**
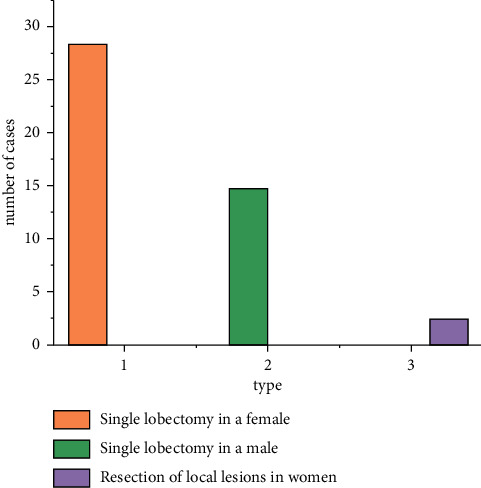
Data graph of patient data.

**Figure 2:**
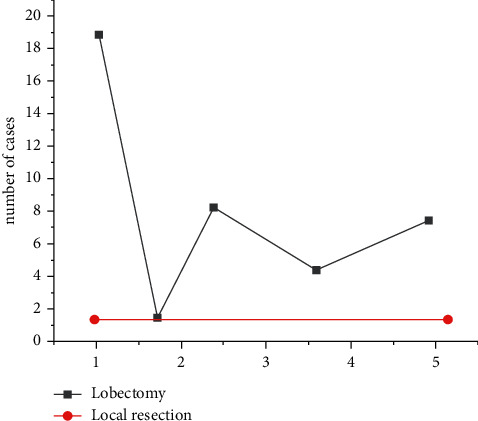
Number of surgical types.

From January to December 2014, 42 patients (15 males and 27 females, aged 33–69 years) underwent lobectomy with the Da Vinci robot system in a chest hospital. The patient's lung shadow, lobulated with uneven density, was found in physical examination or X-ray film for other diseases, and malignant tumor was suspected. After admission, all preoperative examinations were completed, with negative tracheoscopy, normal lung function indicators, normal electrocardiogram, abdominal B ultrasound, head MRI, and whole-body bone scan. No local lymph node or distant lymph node metastasis was found, and all functions were able to tolerate surgery. Preoperative preparation of respiratory tract is perfect, and effective exercise of respiratory function can be carried out [10]. All patients had no thoracic surgery history, no history of malignant tumor, no metastasis was excluded, and no surgical contraindications. As shown in Figure 1, 40 patients underwent unilobectomy, including 19 cases of right upper lobe resection, 2 cases of right middle lobe resection, 8 cases of lower lobe resection, 4 cases of left upper lobe resection, 7 cases of left lower lobe resection, and 2 cases of local lesions of single lung resection. One case of left lower lobe wedge cutting accounts for one quarter of the lung, and one case of right lower lobe wedge cutting accounts for about one third of the lung. The type is shown in Figure 2.

### 2.2. Surgical Methods

Intravenous + complex anesthesia, double lumen endotracheal intubation, single lung ventilation, and right internal jugular vein catheter. The patient was in the 90° decubitus position on the healthy side, with the arm slightly bent on the healthy side, and the affected arm extended forward and upward to fully expose the chest. The waist bridge of the operating table was raised slightly higher than the hip, presenting a folding knife position (about 30° below the waist), providing more operating space for the robotic arm and instrument activities [11]. A 12 mm incision was made in the midaxillary line of the operative side as the observation hole of the robot. Under the guidance of the lens and the monitoring of the display screen, two operation holes of the robotic arm were made, respectively. Another auxiliary hole was made near the operation hole, and a protective sleeve was placed to facilitate the use of the attractor and surgical instruments [12]. Under the guidance of the surgeon, the patient with self-examination completed was pushed into the flat car, which was slowly pushed into the designated position from the cephalic side of the patient, and the lens arm and 2 mechanical arms were assembled (no. 1 arm electric coagulation hook and no. 2 arm Cadiere forceps). The chief surgeon of the operation console explored the thorax without adhesion and satisfied with the collapse of the lung lobe. After the lesion was found, the pulmonary hilum structure was dissected in fine detail. The pulmonary vascular and bronchial structures were treated safely with EC45A, and the lobotomy and systematic mediastinal lymph node dissection were performed. After careful hemostasis, the chief surgeon of the surgeon's console confirmed that there was no bleeding in the chest cavity, the assistant on the table rinsed the chest cavity with sterile injection water, and the anesthesiologist inflated the lungs to confirm that there was no air leakage in the bronchial stump and the rough surface of the lung. The patient's flat carriage was removed, the assistant on the table placed a chest drainage tube, checked, and closed each operation hole, and the operation was finished [13].

## 3. Results and Analysis

### 3.1. Results

There were 42 patients with Da Vinci robot-assisted lobectomy, the operation time was 62–225 min, the blood loss was 70–300 ml, there was no intraoperative blood transfusion, the operation center rate was maintained at 60–100 times/min, the blood pressure was maintained at 90–140/60–90 mmHg, and no patient was transferred to thoracotomy. Among them, 2 patients underwent robotic wedge resection first. The robotic lobectomy was performed after the freezing results confirmed the malignant tumor. After surgery, all patients woke up in the anesthesia recovery room. After waking up, all indicators were qualified (normal blood gas indicators, restored muscle tension, and protective reflex), and vital signs were stable; they returned to the intensive care unit (ward). The mean postoperative hospital stay was (5 ± 2.5) d. The average indwelling time of the chest tube was (4.0 ± 1.9) d. 24 h after surgery, the average volume of chest drainage was (200.4 ± 187.7) mL. Postoperative pain was relatively light, no infection, and the wound was beautiful. All patients were cured and discharged successfully. The intraoperative indicators of the patients are given in Table 1.

### 3.2. Preoperative Visit

#### 3.2.1. Da Vinci Robotic Surgery for Psychological Nursing is a New Minimally Invasive Surgical Method

Patients lack understanding of surgery, and there are concerns and worries about surgery, while patients' emotions and psychology play a very important role in postoperative rehabilitation and treatment of patients. Communicate with patients, understand their inner thoughts, evaluate their physical and psychological states, and patiently and carefully introduce the characteristics and safety of the Da Vinci surgical robot system to patients and their families, as well as the procedures on the day of surgery and the areas needing cooperation during surgery. In the process of communication, patients' inner questions are answered in a timely manner, their tension is eliminated, and patients are psychologically prepared for the smooth operation, so as to enhance their sense of security and trust, so that they can receive surgery in the best physical and mental state [14].

#### 3.2.2. Patient Preparation

One day before surgery, the operating room nurse entered the ward to understand the patient's history, history of present illness, and routine examination indicators. Inform the patient of the importance of fasting and abstaining from drinking in the night before surgery. Patients who have a history of smoking should know when to quit smoking and prepare their respiratory tract. Patients should be informed of the importance of exercise of respiratory function. Before surgery, patients should learn lip constriction breathing, abdominal breathing, whole body relaxation training, and deep inspiratory training. After surgery, patients should learn to cough and expectorate effectively, get out of bed early if the condition allows, and practice deep and slow breathing to promote lung expansion [15].

### 3.3. Preoperative Preparation

#### 3.3.1. Preparation for Itinerant Nurses

One day before surgery, the three components of the surgical robot—surgeon's console, patient flatbed, and camera system flatbed—were tested by professionally trained operating room nurses to ensure that it was in a good standby state. According to the operation needs, adjust the patient flat car position; at the same time, according to the custom of surgeons, prepare special surgical instruments for Da Vinci robot surgery. In case of any special circumstances or faults that cannot be eliminated, contact the head nurse in time. The head nurse will communicate with the robotics department and inform the surgeons of the surgery group. On the day of the operation, the visiting nurse connected the red, green, and blue optical cables again, and the monopole and bipolar electric coagulation foot control the connection line and power line to ensure that the connection is tight and correct, press the “POWER” button to power on, according to the screen prompt, press the “HOME” button to do home, and after all the startup tests passed, inform the operation patient. Lung instruments, disposable dressing kits, washbasin, surgical instruments commonly used for endoscopy, Da Vinci's special surgical instruments (Cadiere grips, electric coagulation hooks, ultrasonic knives, needle holders, and hemolock forceps), and iodophor were prepared with objects [16].

#### 3.3.2. Preparation of Instrument Nurses

The instrument nurse washed her hands 30 min in advance and went on the operating table. Besides checking the conventional surgical dressings, surgical instruments, and stitches, the sterile protective cover of the robot instrument arm, lens arm, and instrument arm should be completed in turn. With the cooperation of the touring nurse, the instrument nurse completed the white balance and focal length of the 30°endoscope and the endoscope calibration specially configured for the system to ensure the perfect match between the endoscope calibrator and the target image, so as to ensure the safe and smooth operation [17].

### 3.4. Intraoperative Coordination

#### 3.4.1. Cooperation of the Instrument Nurse

After the surgeon's routine disinfection of the surgical area, the incision was covered with surgical film, and the outer incision was covered with disposable sterile surgical rib sheet after the surgical towel was spread. Before the operation, the end of the connecting wires of the instruments required for the operation should be handed over to the visiting nurse, and the other end should be properly fixed on the operating table. Assist the surgeon to make an auxiliary hole and an observation hole with a diameter of 12 mm, respectively, and place a protective sleeve in the auxiliary hole to facilitate the access of surgical instruments and suction devices. A disposable puncture device was placed in the observation hole, and CO_2_ gas was injected into the chest cavity at a pressure of 8 mmHg (0.133 kPa) and kept at 6–8 L/min to cause the pulmonary lobe to collapse. An endoscope was placed in the observation hole to observe whether the patient had adhesions and effusion in the chest cavity. Under the guidance of endoscope, the puncture device of no. 1 arm and no. 2 arm were, respectively, placed, and the connection between the Da Vinci robot patient flat car and the puncture device on the patient's incision and the installation of the corresponding instruments (no. 1 arm electric coagulation hook, no. 2 arm Cadiere forceps) were completed. During the operation, the instrument nurse should be familiar with the operation steps, closely observe the operation process displayed on the monitor, clean, replace, and install the surgical instruments in time, and cooperate with the doctors on the table. The instrument nurse should always pay attention to the color signal on the display screen and the arm of each instrument to solve problems in time [18]. When the endoscope lens is blurred, immerse the lens in hot water of about 80°or wipe it with iodophor, and wipe it with dry gauze to ensure clear vision and easy operation, so as to shorten the operation time.

#### 3.4.2. Cooperation of Visiting Nurses

After the operation, the visiting nurse adjusted the pressure and flow of the pneumoperitoneum machine according to the doctor's needs and did a good job in connecting and debugging the instruments. After the surgeon completes incision selection and puncture device placement, the visiting nurse pushes the patient's flat cart from the cephalic direction to the appropriate designated location according to the physician's guidance and navigation. After the patient is connected by the surgeon, the roving nurse makes the registration on the registration form of the robot surgery. During surgery, it is necessary to observe instrument usage in the rooms, especially the pneumoperitoneum machine, the value of pressure and flow rate, positive pressure have an impact on cardiac function in patients with chest, closely observe the patient's heart rate, blood pressure, and oxygen saturation, midway stop pneumoperitoneum machine when necessary, the patient vital signs stable to continue operation, and save items and equipment ready for patients. Due to the anatomical structure of the left upper lobotomy assisted by Leonardo Da Vinci, it is difficult to separate the blood vessels in surgery, and the preparation of materials before surgery needs to be more adequate. In case of emergency, timely supply can be guaranteed.

### 3.5. Postoperative Management

After the operation, the instrument nurse assisted the physician in placing the drainage tube and closing the incision. The instrument nurse and the visiting nurse count the conventional instruments, dressings, and Da Vinci robot special instruments together. Itinerant nurses make registration form of robotic surgery patients, models of surgical instruments, frequency of use, and registration. After the operation, the itinerant nurse disassembled the red, green, and blue cables, respectively, and cleaned the external parts of the three cables and the electrocoagulation connection cables with a soft cloth to keep the cables neat and clean without distortion [19]. Soak endoscope tip in enzyme, rinse, and dry with running water. At the end of the robot surgical instrument, gently scrub the outside of the instrument with a soft brush and alternately rinse the main flushing port, the second flushing port, and the third flushing port with a high-pressure water gun and syringe for at least 20 seconds each time until clear water flows out. Scrub the outside, top, and wrist repeatedly. Running water rinses the outside, top, wrist, and shaft of the device into the housing. Hold the end of the device upward, empty all moisture, and dry the outside of the device with a nonfuzz cloth, blow dry the moisture in the pores with a high-pressure air gun, and lubricate the end and wrist with a neutral pH instrument lubricant. Finally, the surgical instruments were sterilized by the plasma sterilization system. Postoperative nursing satisfaction is given in Table 2.

## 4. Conclusions

In this study, Da Vinci robot-assisted lobectomy was proposed. 42 patients (15 males and 27 females aged 33–69 years old) underwent lobectomy with the Da Vinci robot system in a chest hospital from January to December 2014, and a series of postoperative nursing was performed. The surgical results showed that 42 patients with Da Vinci robot-assisted lobectomy had operation time of 62–225 min, blood loss of 70–300 mL, no intraoperative blood transfusion, operation center rate of 60–100 times/min, and blood pressure of 90–140/60–90 mmHg. None of the patients were transferred to thoracotomy. Among them, 2 patients underwent robotic wedge resection first, and then, robotic lobectomy was performed after malignant tumor was confirmed by freezing results. After surgery, all patients woke up in the anesthesia recovery room. After waking up, all indicators were qualified (normal blood gas indicators, restored muscle tension, and protective reflex), and vital signs were stable; they returned to the intensive care unit (ward). The mean postoperative hospital stay was (5 ± 2.5) d. The average indwelling time of the chest tube was (4.0 ± 1.9) d. 24 h after surgery, the average volume of chest drainage was (200.4 ± 187.7) mL. The postoperative pain was relatively light, no infection, and the wound was beautiful. Robot-assisted lobectomy is a new technique with advantages of less trauma, less pain, faster recovery, and safer and more thorough lymph node dissection. Da Vinci robotic surgery is a new and advanced minimally invasive technique in thoracic surgery, which has been gradually popularized and applied in clinics. Currently, lobectomy is an important method for the treatment of patients with early lung cancer. Although Da Vinci robot-assisted surgery can achieve the minimally invasive treatment effect, it still causes certain trauma to patients. In the future, robot-assisted surgery technology will be further studied.

## Figures and Tables

**Table 1:** Data table of intraoperative indicators.

Group	Surgical time	Amount of bleeding	Heart rate maintenance	BP maintenance
Female	78–189	80–260	75–98	90–140
Male	96–200	70–287	60–100	60–90

**Table 2:** Postoperative nursing evaluation of patients.

Group	Number of cases	Great satisfaction	Satisfactory	Dissatisfaction
Man	15	10	5	0
Woman	27	23	4	0

## Data Availability

The data used to support the findings of this study are available from the corresponding author upon request.
